# Principles of oncology taught in a one-week course

**DOI:** 10.1007/s00432-023-05377-8

**Published:** 2023-09-26

**Authors:** Matthias Oettle, Marcel Büttner, Marie Forster, Laura Gajdi, Johannes Mücke, Alexander Nieto, Sonja Heuser, Johanna Huber, Franziska Walter, Stefanie Corradini, Maximilian Niyazi, Claus Belka, Martin Dreyling, Martin R. Fischer, Daniel F. Fleischmann

**Affiliations:** 1 Institute of Medical Education, University Hospital, LMU Munich, Munich, Germany; 2 Department of Medicine III, University Hospital, LMU Munich, Munich, Germany; 3 Department of Radiation Oncology, University Hospital, LMU Munich, Munich, Germany; 4 German Cancer Consortium (DKTK), Partner Site Munich, Munich, Germany; 5 German Cancer Research Center (DKFZ), Heidelberg, Germany; 6 Bavarian Cancer Research Center (BZKF), Munich, Germany

**Keywords:** Oncology, Education, One-week course

## Abstract

**Background:**

Growing challenges in oncology require evolving educational methods and content. International efforts to reform oncology education are underway. Hands-on, interdisciplinary, and compact course formats have shown great effectiveness in the education of medical students. Our aim was to establish a new interdisciplinary one-week course on the principles of oncology using state-of-the-art teaching methods.

**Methods:**

In an initial survey, medical students of LMU Munich were questioned about their current level of knowledge on the principles of oncology. In a second two-stage survey, the increase in knowledge resulting from our recently established interdisciplinary one-week course was determined.

**Results:**

The medical students’ knowledge of clinically important oncological topics, such as the diagnostic workup and interdisciplinary treatment options, showed a need for improvement. Knowledge of the major oncological entities was also in an expandable state. By attending the one-week course on the principles of oncology, students improved their expertise in all areas of the clinical workup in oncology and had the opportunity to close previous knowledge gaps. In addition, students were able to gain more in-depth clinical knowledge on the most common oncological entities.

**Conclusion:**

The interdisciplinary one-week course on the principles of oncology proved to be an effective teaching method to expand the knowledge of the future physicians to an appropriate level. With its innovative and interdisciplinary approach, the one-week course could be used as a showcase project for the ongoing development of medical education in Germany.

## Introduction

Oncology education shows low levels of connectivity within oncology specialties at many universities around the world (Kanan et al. 2022), whereas in clinical practice, the care of oncology patients is provided on an interdisciplinary basis. Therefore, there is a clear need and demand for improvement towards compact oncology teaching by interdisciplinary teams (Kanan et al. 2022; Mäurer et al. 2023). Study programmes at other universities have shown that oncology education can successfully be integrated early on during undergraduate medical studies (Manirakiza et al. 2020; Rhodin et al. 2020; Lütgendorf-Caucig et al. 2017). As a result, medical students can gain a better understanding of real-world clinical challenges in oncology, such as choosing the best treatment options of medical oncology, oncological surgery, and radiation oncology. Combining classes on basic sciences and clinical hands-on teaching is an effective way to learn disease pathogenesis at the cellular, organ, and patient levels (Brooks et al. 2017). The European Society for Medical Oncology and the American Society of Clinical Oncology already recommend the standardisation of residency training within oncology (Dittrich et al. 2016). Therefore, it is important to further standardise and structure oncology education for medical students as well.

Medical courses taught in an interdisciplinary manner and using digital teaching methods have been the most successful educational formats in previous studies (Back et al. 2016; Dapper et al. 2021) and have been shown to be particularly beneficial to students (Dombrowski et al. 2018; Berman et al. 2016; Crowther and Baillie 2016; Bi et al. 2019). Therefore, new teaching methods are needed (Schwartz et al. 2007; Ilkiw et al. 2017; Dickinson et al. 2018) with a focus on case-based learning (Thistlethwaite et al. 2012; Hassoulas et al. 2017; Gade and Chari 2013; Preeti et al. 2013; Bonney 2015; Hofsten et al. 2010; Lee Chin et al. 2014). Compact course formats in an interdisciplinary team have been found to be very effective in teaching (Kanan et al. 2022). Previous work has shown that one-week intensified courses are well suited for oncology education (Scheide et al. 2020).

Our aim was to establish a new interdisciplinary one-week block course on the principles of oncology using state-of-the-art teaching methods. The focus was to provide each medical student with a deep basic knowledge of the major oncological entities. The increase in knowledge of the medical students was assessed through surveys of medical students prior to and at the end of the one-week block course.

## Methods

### Study setting and participants

We conducted two surveys: In the first survey, participants from both preclinical and clinical study sections were questioned on their knowledge of oncology topics. To be eligible, participants had to be enrolled at the Medical Faculty of the LMU.

The second survey was conducted in February 2022 among medical students enrolled in the first clinical semester, who voluntarily participated in the newly designed one-week block course, and assessed their knowledge before and after the course (for chronological project overview see Supplementary Fig. 1).

### Questionnaire design and preparation

According to the guidelines of the ethics committee of the Medical Faculty of LMU, the data was collected and analysed anonymously. All questionnaires were prepared using the evasys software (V8.2, evasys GmbH, Lüneburg, Germany).

The first survey was conducted with a questionnaire with 56 items, including two single-choice questions, three open-ended questions, and 51 questions with a five-point scale. In the second survey, the baseline and the end-of-course questionnaires each contained 53 items, including two single-choice questions and 51 questions with a five-point scale (for complete questionnaires, see Supplementary Appendix).

Single-choice questions were used to differentiate participants according to their study progress, open-ended questions were used to capture individual student requests and suggestions (data not shown), and five-point scales were used to record the levels of knowledge and of previous contact with the various disciplines. Accordingly, a higher value on the five-point scale indicates a higher level of knowledge or a higher degree of previous contact with oncological topics.

### Survey implementation

The questionnaires of the first survey were distributed to all medical students of LMU via email and the e-learning platform Moodle (med.moodle.elearning.lmu.de) on 23rd April 2021. After being accessible for 20 weeks, the survey was closed with 237 evaluable entries.

**Fig. 1:**
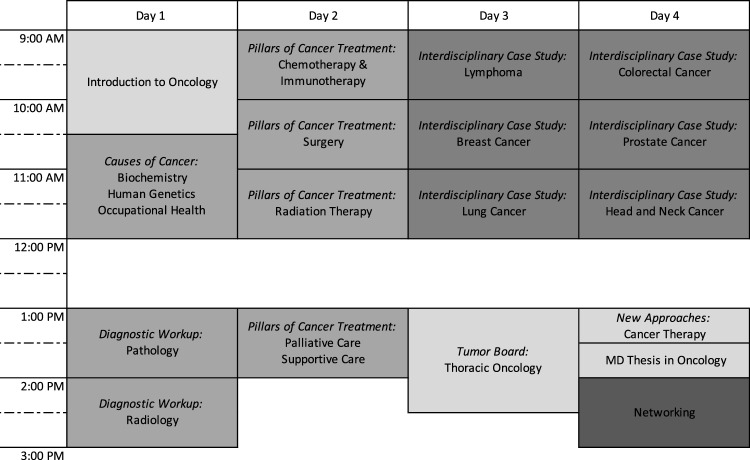
Timetable of the one-week block course on the principles of oncology. Light grey: key lectures, medium grey: principles of oncology, dark grey: interdisciplinary case studies, very dark grey: voluntary networking event

In the second survey, all students who participated in a one-week block course on the basics of oncology were sent an entry questionnaire prior to and at the beginning of the first lecture and an exit questionnaire after the final lecture and subsequently by email. The baseline questionnaire, which was available from 14th February until 18th February 2022, was fully evaluable for 38/40 participants (95%). The end-of-course questionnaire, available from 22nd February until 4th March 2022, was completed by 35 students (Fig. 1).

### Project overview

The one-week block course on the principles of oncology was developed following the six-step-approach to curricular development in medical education by Thomas et al. (Thomas et al. 2015):

1. Problem identification and general needs assessment
  Multiple interviews and discussions among faculty representatives and student council members revealed potential for improvement in the curriculum. Consequently, learning objectives, the principles of oncology and six core cancer entities were defined.
2. Targeted needs assessment
  In order to quantify the need for restructuring, the first survey was conducted as described above.
3. Goals and objectives
  The goals of the project were to develop an interdisciplinary, longitudinal teaching concept for oncology and to implement a one-week block course permanently in the first clinical semester of the Medical Curriculum Munich (MeCuM). Further aims of the project were the consolidation of the students’ knowledge on the principles of oncology from the preclinical study phase and the application of competency-based learning as defined in the “National Competence Based Catalogues of Learning Objectives for Undergraduate Medical Education” (NKLM) (Fischer et al. 2015).
4. Educational strategies
  The one-week block course on the principles of oncology was divided into three sections (see Figure 2). The first part included courses on the basic aetiology, epidemiology, and diagnosis of cancer. The second part outlined the variety of treatment strategies, followed by the third part, in which core tumour entities were introduced and exemplified through case studies. Various teaching formats such as co-teaching, inverted classroom, case studies, and didactically adapted tumour boards were included. Extensive e-learning with virtual patient cases complemented the one-week course.

**Fig. 2:**
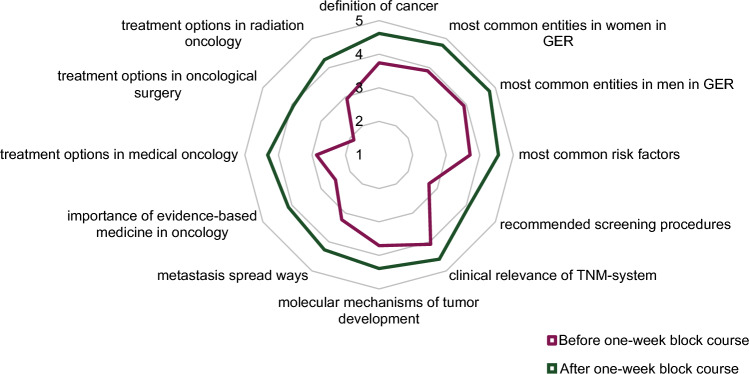
Knowledge on principles of oncology before and after one-week block course. Radar plot with items from the 2nd survey. Numbers correspond to five-point scale and are mean values. For measuring the level of knowledge, a five-point scale was used (1 [very little knowledge] to 5 [very much knowledge]). “GER” is short for Germany
5. Implementation
  The one-week block course on the principles of oncology was designed and planned from 2018 to 2022 and took place over four days in February 2022 (see Figure 1). The course consisted of a total of 17 sessions, ranging in length between 15 and 90 minutes. The sessions were given by 25 lecturers from 14 different disciplines, including biochemistry, ENT, gynaecology, haematology/oncology, human genetics, occupational health, palliative care, pathology, pharmacology, pneumology, radiation therapy, radiology, urology, and visceral surgery division. Due to restrictions imposed by the SARS-CoV-2 pandemic, all sessions took place online on the Zoom video conferencing platform (Zoom Video Communications, V5, San Jose, CA).
6. Evaluation and feedback
  The evaluation of the one-week block course on the principles of oncology was conducted with a two-part survey as described above.

### Statistical analysis

The data was collected with evasys (evasys GmbH, Lüneburg, Germany), prepared using Microsoft Excel (Mac version 16.64, Microsoft, Redmond, WA, USA), and statistically analysed with GraphPad Prism (V9, GraphPad Software, San Diego, CA, USA) as well as IBM SPSS Statistics (V28, IBM Corp., Armonk, NY, USA).

To assess prior experience, students were asked about the level of previous interaction with various disciplines. The five-point scale for this section ranged from 1 “no interaction at all” to 5 “heavily involved”. In the assessment of the existing level of knowledge, ratings 1 and 2 were evaluated as “very little knowledge” and “little knowledge”, respectively, rating 3 was evaluated as “medium knowledge”, and ratings 4 and 5 were evaluated as “rather much knowledge” and “very much knowledge”, respectively. To facilitate understanding of the data presentation, some of the 53 items were grouped into categories: four questions each on “epidemiology and prevention of cancer” and “cancer pathology and its clinical relevance” were grouped together, as were six questions on each of six entities. The results tested negative for normal distribution; therefore, the Mann–Whitney U test was used for nonparametric comparisons. Due to multiple testing, the significance level was Bonferroni adjusted to α = 0.00417 for all tests. Percentages were rounded to the nearest integer.

## Results

### Targeted needs assessment

A total of 125 preclinical students and 112 clinical students participated in the first survey. The preclinical students had completed an average of 3.0 semesters, while the clinical students had completed an average of 6.7 semesters at the time of the survey. All students reported to have had little to moderate prior involvement with medical oncology, radiation oncology, and oncological surgery (see Table 1).

**Table 1:** Self-reported knowledge of oncology

Student characteristics	Preclinical years Mean (St. Dev)	Clinical years Mean (St. Dev)	Total Mean (St. Dev.)
*N*, %	125, 53%	112, 47%	237
# Semesters	3.0 (1.0)	6.7 (1.0)	4.6 (2.1)
*Previous contact with*			
Medical oncology	2.1 (0.9)	3.4 (1.1)	2.6 (1.0)
Oncological surgery	1.3 (0.6)	2.7 (1.0)	1.9 (1.0)
Radiation oncology	1.3 (0.6)	2.8 (1.1)	2.0 (1.1)
*Knowledge level*			
*Basic knowledge about*			
Definition of cancer	3.8 (1.1)	4.2 (0.8)	4.0 (1.0)
Epidemiology and prevention of cancer	3.2 (1.2)	4.0 (1.0)	3.6 (1.2)
Cancer pathology and its clinical relevance	2.2 (1.3)	3.6 (1.2)	2.8 (1.4)
Treatment options in medical oncology	2.6 (1.2)	3.4 (1.1)	2.9 (1.2)
Treatment options in radiation oncology	1.9 (1.1)	2.7 (1.0)	2.3 (1.1)
Treatment options in oncological surgery	1.8 (1.0)	3.0 (1.1)	2.3 (1.2)
*Entity-specific knowledge about*			
Lung cancer	1.9 (1.1)	2.9 (1.2)	2.3 (1.2)
Breast cancer	2.5 (1.3)	3.2 (1.2)	2.8 (1.3)
Prostate cancer	2.0 (1.1)	3.2 (1.3)	2.5 (1.3)
Rectal cancer	1.7 (1.0)	2.8 (1.2)	2.2 (1.3)
Lymphoma	1.7 (1.1)	2.8 (1.2)	2.2 (1.3)
Pharyngeal cancer	1.3 (0.7)	1.8 (1.1)	1.5 (0.9)

All values are mean values (St. Dev.), unless otherwise specified. For measuring the previous points of contact with different specialities and the level of knowledge, a five-point scale was used (1 [no contact at all] to 5 [strongly involved]; 1 [very little knowledge] to 5 [very much knowledge])

Regarding basic knowledge of oncology, students overall reported knowing “medium” to “rather much” about the definition of cancer, as well as on the epidemiology and prevention of cancer. Preclinical students reported mean scores of 3.8 (± 1.1) and 3.2 (± 1.2), while clinical students reported higher mean scores of 4.2 (± 0.8) and 4.0 (± 1.0), respectively. Regarding pathology and treatment options in medical oncology, radiation oncology, and oncological surgery, both preclinical and clinical students reported only “medium” knowledge (overall 2.8 (± 1.4), 2.9 (± 1.2), 2.3 (± 1.1), and 2.3 (± 1.1) points on the five-point scale). The average increase in knowledge from preclinical to clinical study section ranged only between 0.4 and 1.3 points. For example, concerning radiation oncology treatment options, clinical students reported only 0.8 higher mean values on the five-point scale than students in the preclinical Sect. (1.9 (± 1.1) vs. 2.7 (± 1.0) reported mean scale value preclinical vs. clinical students) (see Table 1; for full dataset see Supplementary Appendix).

In terms of knowledge about specific entities (lung cancer, breast cancer, prostate cancer, rectal cancer, lymphoma, pharyngeal cancer), students overall reported a mean knowledge level of below 3.0, indicating “very little”, “little”, or “medium” knowledge. Students of the preclinical semesters reported less knowledge than students of the clinical semesters. Notably out of all items queried the maximum increase in knowledge from preclinical to clinical study section was 1.3 points (mean on the five-point scale). In contrast knowledge gain for the most frequent cancer entities was low, e.g. for breast carcinoma an improvement of only 0.7 points was detected (see Table 1; for full dataset see Supplementary Appendix).

### Evaluation of the one-week block course on the principles of oncology

Of 40 medical students enrolled in the one-week block course, 38 participated in the first part of the survey prior to the course and 35 participated in the second part after completion of the course. All students reported a significant increase in knowledge in all areas surveyed (see Table 2).

**Table 2:** Evaluation of the one-week block course on the principles of oncology

Student characteristics	Before one-week block course		After one-week block course		Mann–Whitney *U* test			
	Mean (St. Dev)	Mean rank	Mean (St. Dev)	Mean rank	Mann–Whitney *U*	Z	*p value*	Cohen’s d
*N*	38	–	35	–	–	–		–
# semesters in clinical stage	1.1 (0.5)	-	1.1 (0.5)	–	–	–		–
*Previous contact with*								
Medical oncology	2.7 (1.1)	–	–	–	–	–		–
Oncological surgery	1.4 (0.9)	–	–	–	–	–		–
Radiation oncology	3.3 (0.7)	–	–	–	–	–		–
*Knowledge level*								
*Basic knowledge about*								
Definition of cancer	3.7 (0.9)	26.9	4.6 (0.8)	48.0	281.5	– 4.6	*p* < *0.001*	– 1.1
Epidemiology and prevention of cancer	3.6 (0.9)	103.0	4.6 (0.8)	192.4	4081.0	– 9.6	*p* < *0.001*	– 1.1
Cancer pathology and its clinical relevance	3.4 (1.1)	109.8	4.4 (0.9)	185.6	5059.0	– 8.0	*p* < *0.001*	– 0.9
Treatment options in medical oncology	2.9 (1.1)	24.6	4.3 (0.9)	50.5	192.0	– 5.4	*p* < *0.001*	– 1.3
Treatment options in oncological surgery	1.9 (0.9)	21.8	4.0 (0.9)	53.5	86.5	– 6.5	*p* < *0.001*	– 1.5
Treatment options in radiation oncology	2.9 (0.9)	24.5	4.3 (0.9)	50.6	189.0	– 5.4	*p* < *0.001*	– 1.3
*Entity-specific knowledge about*								
Lung cancer	2.3 (0.9)	128.6	4.2 (0.9)	307.7	3792.5	– 15.3	*p* < *0.001*	– 1.5
Breast cancer	3.0 (1.1)	138.4	4.5 (0.8)	296.4	6048.0	– 13.7	*p* < *0.001*	– 1.3
Prostate cancer	2.7 (1.1)	134.6	4.4 (0.9)	299.0	5303.0	– 14.2	*p* < *0.001*	– 1.4
Rectal cancer	2.4 (1.0)	126.3	4.3 (0.9)	300.8	3842.5	– 15.1	*p* < *0.001*	– 1.4
Lymphoma	2.4 (1.0)	137.4	4.0 (1.0)	289.2	6244.5	– 13.1	*p* < *0.001*	– 1.3
Tongue cancer	1.6 (0.9)	123.8	4.1 (1.0)	308.1	2936.0	– 16.0	*p* < *0.001*	– 1.6

All values are mean values (St. Dev.), unless otherwise specified. For measuring the previous points of contact with different specialities and the level of knowledge, a five-point scale was used (1 [no point of contact at all] to 5 [strongly involved]; 1 [very little knowledge] to 5 [very much knowledge]). Significance level was Bonferroni adjusted to α = 0.00417, *p* value ≤ 0.001 (significant). Cohen’s |d|≥ 0.8 (large effect size)

Concerning the definition of cancer, epidemiology, and prevention of cancer, as well as cancer pathology, students indicated “medium” to “rather much” knowledge even before the one-week course. After the one-week block course, knowledge increased in mean by 0.9 to 1.0 points on the five-point scale, indicating that students now reached “rather much” to “very much” knowledge.

When evaluating the treatment options in medical oncology, radiation oncology, and oncological surgery, students reported only little to moderate prior knowledge. After completion of the one-week block course, the knowledge of the different cancer treatment options increased by 1.4 to 2.0 points (mean on the five-point scale). Similarly, knowledge on surgical treatment options increased from 1.9 (± 0.9) to 4.0 (± 0.9) level of knowledge before vs. after the course.

Tumour-specific knowledge showed the greatest increase after the one-week course, with mean values between 1.6 and 3.0 vs 3.9 to 4.5 on the five-point scale, prior vs. after the course, respectively. The knowledge on tongue cancer for instance was reported 1.6 (± 0.9) vs. 4.1 (± 1.0) (mean on the five-point scale).

After attending the one-week block course, students improved their knowledge in all areas surveyed, with the largest increase in the areas of “recommended screening procedures” from (in mean 2.8–4.1 points), “importance of evidence-based medicine in oncology” (2.5–4.3 points), “treatment options in medical oncology” (2.9–4.3 points), and “treatment options in oncological surgery” (1.9–4.0 points) (see Fig. 2 or Supplementary Appendix).

**Fig. 3:**
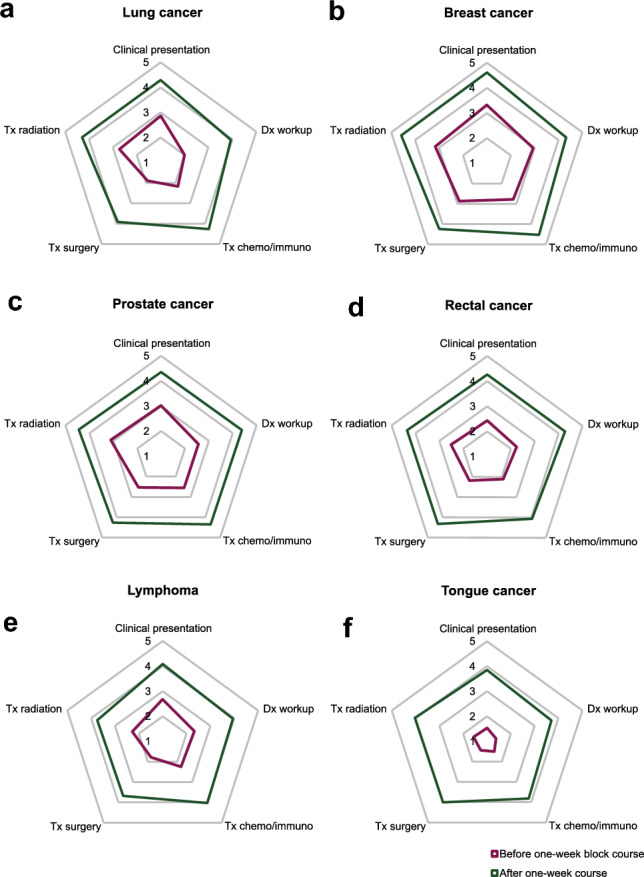
Knowledge of core tumour entities before and after one-week course. Radar plot with items from the 2nd survey. Numbers correspond to five-point scale and are mean values. For measuring the level of knowledge, a five-point scale was used (1 [very little knowledge] to 5 [very much knowledge]). “Dx” is short for “diagnostic”, “Tx” for “therapeutic options”

Across all six cancer entities taught, the course enabled students to improve their knowledge on clinical presentation, diagnostic workup and treatment options in medical oncology, radiation oncology, and oncological surgery. The highest increases in knowledge were reported especially for tongue, rectal, and lung cancers, respectively, in diagnostic workup strategies and medical and surgical treatment options (see Fig. 3).

## Discussion

As the global demographic burden of cancer is steadily increasing, oncology takes on one of the most important roles in medicine today (Kocarnik et al. 2022), which has to be reflected in medical curricula. We strongly believe that emphasis on teaching basic oncology skills to medical students will improve their preparation in a world with an increasing number of cancer patients within each of the medical specialties (Bravery et al. 2020; Al Suwayri 2018; DeNunzio et al. 2013).

In addition, oncology is becoming increasingly complex. Reasons for this include the increasing use of genomic profiling towards precision oncology (Mateo et al. 2022), the increase in subspecialized care, rapid technological advances, and the increasing emphasis on cost efficiency in hospital management (Pershing and Fuchs 2013). These challenges need to be met by specifically trained medical oncology staff, which begins with improved oncology education during medical study (Pershing and Fuchs 2013; Loyola 2010).

The apparent need for improvement in oncology education and its teaching prompted us to investigate the level of oncological knowledge of preclinical and clinical students. The first survey included students from the first to the last semester of our medical faculty, was intended to serve as one of the bases for designing the one-week block course on the principles of oncology.

While most of the preclinical and clinical students knew about the definition of cancer and had moderate to good knowledge of basic epidemiology, they reported little or very little knowledge in other areas of oncology (see Table 1). Especially, regarding screening tests for the most common cancer entities, students reported their knowledge as low as 2.6 and 3.3 in mean on the five-point scale in the preclinical as well as in the clinical cohort, respectively.

With regard to the therapeutic options in treatment of cancer, more than a fifth of the clinical students reported to have only a basic knowledge. In terms of entity-specific knowledge, clinical students reported only moderate knowledge of even the most common entities such as breast cancer, prostate cancer, and lung cancer.

In this paper, we report rather low levels of self-reported oncology knowledge, even among clinical students. While we lack paired data on the true oncological knowledge as assessed by standardised tests, our results clearly indicate that there is an urgent need to re-structure oncology education.

Thus, we developed a one-week block course on the principles of oncology in the first clinical year in order to teach the essentials of oncology at an early educational stage and to introduce the most common tumour entities. This one-week block course would be the first milestone towards a longitudinal oncology curriculum at LMU Munich, which will accompany student along all their study of medicine.

The new National Competency-Based Learning Catalogue for Medicine 2.0 (NKLM 2.0) will become a mandatory part of German medical studies and mandates restructuring by 2025. It aims at teaching preclinical basics and clinical knowledge jointly in a longitudinal and interprofessional manner (Ärzteblatt and Modellstudiengänge: Bausteine für ein gutes 2022; Dapper et al. 2022; Ärzteblatt et al. 2022). With our one-week block course on principles of oncology, we strengthened and built bridges between the preclinical and clinical settings. This has been previously described above as a core element of a high-quality medical curriculum (Boeker et al. 2005).

By demonstrating the benefit from a one-week block course regardless of students’ prior knowledge, we were able to confirm previous published data (Boeker et al. 2005; Cecilio-Fernandes et al. 2018), suggesting that a compact course is sufficient to significantly increase self-reported knowledge of oncology topics. In fact, our results prove that the one-week block course is able to increase knowledge level on therapeutic options in cancer treatment by 48% (from 2.9 to 4.3).

In line with these results, we also observed an increase in clinical presentation of different entities, their diagnostic workup, and their therapeutic strategies.

For this course, we employed a variety of modern teaching methods such as an inverted-classroom approach (ICA) via e-learning, interdisciplinary co-teaching, and case-based learning (CBL). The ICA, which allows students to be better prepared for upcoming lectures (Boeker et al. 2005), was implemented via e-learning, as this format has been (Boeker et al. 2005) successfully established in various medical disciplines (Dapper et al. 2022; Boeker et al. 2005; Huber et al. 2021; Röcker et al. 2021).

Co-teaching has been shown to improve the links between preclinical and clinical content (Willey et al. 2018). We used these tools to comprehensively teach the causes of cancer by recruiting a biochemist, a human geneticist, and an occupational health physician. The seminars on frequent core cancer entities were also held by different specialists from the surgical, internal medicine, and radiation oncology departments in order to take advantage of the above-mentioned co-teaching approach.

Case-based learning (CBL) was used as a core component of the one-week block course, as it has been demonstrated to promote critical thinking and clinical problem-solving skills, with significant improvements in knowledge acquisition (Brooks et al. 2017; Berman et al. 2016; Crowther and Baillie 2016; Hassoulas et al. 2017; Gade and Chari 2013; Preeti et al. 2013; Bonney 2015; Hofsten et al. 2010; Lee Chin et al. 2014; Yoo and Park 2015; Dupuis and Persky 2008; Ali et al. 2018; Lee et al. 2013; Ilgüy et al. 2014). In addition to the use of CBL on days 3 and 4 for the entity-specific lectures, it was also an integral part of the virtual tumour board on day 3. In this educational format, the previously acquired knowledge about lung cancer should be applied by students in didactically prepared tumour board cases. Hereby, interdisciplinary oncological decision-making was made accessible to the medical students. Further research is warranted to determine the most efficient teaching methods.

Due to the increasing incidence and complexity of cancer (Ferlay et al. 2018; Wild et al. 2019), continuous adaptation of the medical curricula is required to ensure high-quality education and consequently good patient care in oncology. Our results demonstrate that the need for continuous improvement in oncology education can successfully be addressed through interdisciplinary teaching in a one-week format.

## Data Availability

The data supporting the findings of this study are available within the article and its supplementary materials.

## References

[CR1] Al Suwayri SM (2018). Feasibility and outcomes of oncology teaching for 5th year medical students. J Cancer Educ.

[CR2] Ali M, Han SC, Bilal HSM, Lee S, Kang MJY, Kang BH, Razzaq MA, Amin MB (2018). iCBLS: an interactive case-based learning system for medical education. Int J Med Inform.

[CR3] Deutsches Ärzteblatt. Modellstudiengänge: Bausteine für ein gutes Studium. 2014; https://www.aerzteblatt.de/archiv/152983/Modellstudiengaenge-Bausteine-fuer-ein-gutes-Studium. Accessed Oct 22, 2022.

[CR4] Deutsches Ärzteblatt. Medizinische Fakultäten wollen sich bei Neustrukturierung des Medizinstudiums engagieren. 2019; https://www.aerzteblatt.de/nachrichten/104083/Medizinische-Fakultaeten-wollen-sich-bei-Neustrukturierung-des-Medizinstudiums-engagieren. Accessed Oct 22, 2022.

[CR5] Back DA, Behringer F, Haberstroh N, Ehlers JP, Sostmann K, Peters H (2016). Learning management system and e-learning tools: an experience of medical students' usage and expectations. Int J Med Educ.

[CR6] Berman NB, Durning SJ, Fischer MR, Huwendiek S, Triola MM (2016). The role for virtual patients in the future of medical education. Acad Med.

[CR7] Bi M, Zhao Z, Yang J, Wang Y (2019). Comparison of case-based learning and traditional method in teaching postgraduate students of medical oncology. Med Teach.

[CR8] Boeker M, Müller C, Klar R, Lutterbach J (2005). OncoCase: interdisciplinary case based teaching in Neuro-Oncology based on the campus platform. AMIA Annu Symp Proc.

[CR9] Bonney KM (2015). Case study teaching method improves student performance and perceptions of learning gains. J Microbiol Biol Educ.

[CR10] Bravery BD, Shi K, Nicholls L, Chelvarajah R, Tieu MT, Turner S, Windsor A (2020). Oncology and radiation oncology awareness in final year medical students in Australia and New Zealand. J Cancer Educ.

[CR11] Brooks EG, Thornton JM, Ranheim EA, Fabry Z (2017). Incorporation of autopsy case-based learning into PhD graduate education: a novel approach to bridging the "bench-to-bedside" gap. Hum Pathol.

[CR12] Cecilio-Fernandes D, Aalders WS, de Vries J, Tio RA (2018). The impact of massed and spaced-out curriculum in oncology knowledge acquisition. J Cancer Educ.

[CR13] Crowther E, Baillie S (2016). A method of developing and introducing case-based learning to a preclinical veterinary curriculum. Anat Sci Educ.

[CR14] Dapper H, Wijnen-Meijer M, Rathfelder S, Mosene K, von Kirchbauer I, Bernhardt D, Berberat PO, Combs SE (2021). Radiation oncology as part of medical education-current status and possible digital future prospects. Strahlenther Onkol.

[CR15] Dapper H, Belka C, Bock F, Budach V, Budach W, Christiansen H, Debus J, Distel L, Dunst J, Eckert F (2022). Integration of radiation oncology teaching in medical studies by German medical faculties due to the new licensing regulations: an overview and recommendations of the consortium academic radiation oncology of the German Society for Radiation Oncology (DEGRO). Strahlenther Onkol.

[CR16] DeNunzio NJ, Joseph L, Handal R, Agarwal A, Ahuja D, Hirsch AE (2013). Devising the optimal preclinical oncology curriculum for undergraduate medical students in the United States. J Cancer Educ.

[CR17] Dickinson BL, Lackey W, Sheakley M, Miller L, Jevert S, Shattuck B (2018). Involving a real patient in the design and implementation of case-based learning to engage learners. Adv Physiol Educ.

[CR18] Dittrich C, Kosty M, Jezdic S, Pyle D, Berardi R, Bergh J, El-Saghir N, Lotz JP, Österlund P, Pavlidis N et al (2016) ESMO/ASCO Recommendations for a global curriculum in medical oncology Edition 2016. ESMO Open 1(5):e000097 10.1136/esmoopen-2016-000097 PMC5070299 27843641

[CR19] Dombrowski T, Wrobel C, Dazert S, Volkenstein S (2018). Flipped classroom frameworks improve efficacy in undergraduate practical courses - a quasi-randomized pilot study in otorhinolaryngology. BMC Med Educ.

[CR20] Dupuis RE, Persky AM (2008). Use of case-based learning in a clinical pharmacokinetics course. Am J Pharm Educ.

[CR21] Ferlay J, Colombet M, Soerjomataram I, Dyba T, Randi G, Bettio M, Gavin A, Visser O, Bray F (2018). Cancer incidence and mortality patterns in Europe: Estimates for 40 countries and 25 major cancers in 2018. Eur J Cancer.

[CR22] Fischer MR, Bauer D, Mohn K (2015) Finally finished! National Competence Based Catalogues of Learning Objectives for Undergraduate Medical Education (NKLM) and Dental Education (NKLZ) ready for trial. GMS Z Med Ausbild 32(3):Doc35 10.3205/zma000977 PMC4580444 26677513

[CR23] Gade S, Chari S (2013). Case-based learning in endocrine physiology: an approach toward self-directed learning and the development of soft skills in medical students. Adv Physiol Educ.

[CR24] Hassoulas A, Forty E, Hoskins M, Walters J, Riley S (2017). A case-based medical curriculum for the 21st century: The use of innovative approaches in designing and developing a case on mental health. Med Teach.

[CR25] Hofsten A, Gustafsson C, Häggström E (2010). Case seminars open doors to deeper understanding - Nursing students' experiences of learning. Nurse Educ Today.

[CR26] Huber J, Witti M, Schunk M, Fischer MR, Tolks D (2021). The use of the online Inverted Classroom Model for digital teaching with gamification in medical studies. GMS J Med Educ.

[CR27] Ilgüy M, Ilgüy D, Fişekçioğlu E, Oktay I (2014). Comparison of case-based and lecture-based learning in dental education using the SOLO taxonomy. J Dent Educ.

[CR28] Ilkiw JE, Nelson RW, Watson JL, Conley AJ, Raybould HE, Chigerwe M, Boudreaux K (2017). Curricular revision and reform: the process, what was important, and lessons learned. J Vet Med Educ.

[CR29] Kanan D, Kanan T, Kalyenci N, Nanah AR, Tarbaghia M, Ekmekci B, Çelik S, Öven BB (2022). A successful model for an introductory oncology teaching conference and its impact on preclinical and clinical medical students. JCO Oncol Pract.

[CR30] Kocarnik JM, Compton K, Dean FE, Fu W, Gaw BL, Harvey JD, Henrikson HJ, Lu D, Pennini A, Xu R (2022). Cancer incidence, mortality, years of life lost, years lived with disability, and disability-adjusted life years for 29 cancer Groups from 2010 to 2019: a systematic analysis for the global burden of disease study 2019. JAMA Oncol.

[CR31] Lee BF, Chiu NT, Li CY (2013). Value of case-based learning in a nuclear medicine clerkship. J Am Coll Radiol.

[CR32] Lee Chin K, Ling Yap Y, Leng Lee W, Chang Soh Y (2014). Comparing effectiveness of high-fidelity human patient simulation vs case-based learning in pharmacy education. Am J Pharm Educ.

[CR33] Loyola S (2010). Evidence-based teaching guidelines: transforming knowledge into practice for better outcomes in healthcare. Crit Care Nurs Q.

[CR34] Lütgendorf-Caucig C, Kaiser PA, Machacek A, Waldstein C, Pötter R, Löffler-Stastka H (2017). Vienna Summer School on Oncology: how to teach clinical decision making in a multidisciplinary environment. BMC Med Educ.

[CR35] Manirakiza A, Rubagumya F, Fehr AE, Triedman AS, Greenberg L, Mbabazi G, Ntacyabukura B, Nyagabona S, Maniragaba T, Longombe AN (2020). Oncology training in rwanda: challenges and opportunities for undergraduate medical students (The EDUCAN Project). J Cancer Educ.

[CR36] Mateo J, Steuten L, Aftimos P, André F, Davies M, Garralda E, Geissler J, Husereau D, Martinez-Lopez I, Normanno N (2022). Delivering precision oncology to patients with cancer. Nat Med.

[CR37] Mäurer M, Staudacher J, Meyer R, Mäurer I, Lazaridis L, Müther M, Huber T, Sommer NP, Fleischmann DF, Käsmann L (2023). Importance of interdisciplinarity in modern oncology: results of a national intergroup survey of the Young Oncologists United (YOU). J Cancer Res Clin Oncol.

[CR38] Pershing S, Fuchs VR (2013). Restructuring medical education to meet current and future health care needs. Acad Med.

[CR39] Preeti B, Ashish A, Shriram G (2013). Problem based learning (PBL) - an effective approach to improve learning outcomes in medical teaching. J Clin Diagn Res.

[CR40] Rhodin KE, Hong CS, Olivere LA, Howell EP, Giri VK, Mehta KA, Oyekunle T, Scheri RP, Tong BC, Sosa JA (2020). Implementation of a surgical oncology disparities curriculum for preclinical medical students. J Surg Res.

[CR41] Röcker N, Lottspeich C, Braun LT, Lenzer B, Frey J, Fischer MR, Schmidmaier R (2021). Implementation of self-directed learning within clinical clerkships. GMS J Med Educ.

[CR42] Scheide L, Huber T, Bette S, Nest A, Zimmer C, Berberat PO, Kreiser K (2020). "Imagine Neuro-Oncology"- a one week course with medical and technical students: students' reflections about multidisciplinarity and its practical relevance. J Interprof Care.

[CR43] Schwartz LR, Fernandez R, Kouyoumjian SR, Jones KA, Compton S (2007). A randomized comparison trial of case-based learning versus human patient simulation in medical student education. Acad Emerg Med.

[CR44] Thistlethwaite JE, Davies D, Ekeocha S, Kidd JM, MacDougall C, Matthews P, Purkis J, Clay D (2012) The effectiveness of case-based learning in health professional education. A BEME systematic review: BEME Guide No. 23. Med Teach 34(6):e421–444 10.3109/0142159X.2012.680939 22578051

[CR45] Thomas PA, Kern DE, Hughes MT, Chen BY (2015) Curriculum development for medical education: A six-step approach. Johns Hopkins University Press

[CR46] Wild CP, Espina C, Bauld L, Bonanni B, Brenner H, Brown K, Dillner J, Forman D, Kampman E, Nilbert M (2019). Cancer prevention Europe. Mol Oncol.

[CR47] Willey JM, Lim YS, Kwiatkowski T (2018). Modeling integration: co-teaching basic and clinical sciences medicine in the classroom. Adv Med Educ Pract.

[CR48] Yoo MS, Park HR (2015). Effects of case-based learning on communication skills, problem-solving ability, and learning motivation in nursing students. Nurs Health Sci.

